# Comorbidities, injury severity and complications predict mortality in thoracic trauma

**DOI:** 10.1007/s00068-022-02177-6

**Published:** 2022-12-17

**Authors:** Anne T. Fokkema, Bergros K. Johannesdottir, Klaus Wendt, Rune Haaverstad, Inge H. F. Reininga, Thomas Geisner

**Affiliations:** 1 Department of Trauma Surgery, University of Groningen, University Medical Center Groningen, Hanzeplein 1, P.O. Box 30.001, 9700RB Groningen, The Netherlands; 2 Department of Vascular Surgery, Haukeland University Hospital, Bergen, Norway; 3 Section of Cardiothoracic Surgery, Department of Heart Disease, University of Bergen, Haukeland University Hospital, Bergen, Norway; 4 University of Iceland, Reykjavik, Iceland; 5 Institute of Clinical Science, University of Bergen, The Medical Faculty, Bergen, Norway; 6 Western Norway Trauma Center, Haukeland University Hospital, Bergen, Norway

**Keywords:** Thoracic injuries, Mortality, Triage, Trauma, Comorbidity, Polypharmacy

## Abstract

**Purpose:**

Thoracic trauma accounts for 25–50% of posttraumatic mortality. Data on epidemiology of thoracic trauma in Scandinavia and risk factors for mortality are scarce. This study aims to provide an overview of epidemiology, clinical events and risk factors for mortality of patients with severe thoracic injuries.

**Methods:**

A retrospective study including adult thoracic trauma patients with abbreviated injury scale ≥ 3, between 2009 and 2018 at Haukeland University Hospital was performed. Subgroup analyses were performed for specific patient groups: (1) isolated thoracic trauma, (2) polytrauma without Traumatic Brain Injury (TBI) and (3) polytrauma with TBI. Logistic regression analyses were applied to find risk factors for 30-days mortality. Age, sex, comorbidity polypharmacy score (CPS), trauma and injury severity score (TRISS) and comprehensive complication index (CI) were included in the final model.

**Results:**

Data of 514 patients were analyzed, of which 60 (12%) patients died. Median (IQR) injury severity score (ISS) was 17 (13–27). Data of 463 patients, of which 39 patients died (8%), were included in multivariate analyses. Female sex odds ratio (OR) (2.7, *p* = 0.04), CPS > 9 (OR 4.8; *p* = 0.01), TRISS ≤ 50% (OR 44; *p* < 0.001) and CI ≥ 30 (OR 12.5, *p* < 0.001) were significant risk factors for mortality. Subgroup analyses did not demonstrate other risk factors.

**Conclusion:**

Comorbidities and associated pharmacotherapies, TRISS, female sex, and complications during admission predict in-hospital mortality after thoracic trauma. Current findings might help to recognize patients at risk of an adverse outcome, and thereby prevent complications.

**Trial registration: retrospectively registered:**

The regional committees for medical and health research ethics file number is 2017/293.

**Supplementary Information:**

The online version contains supplementary material available at 10.1007/s00068-022-02177-6.

## Introduction

The thorax is a body region that is frequently and severely injured. Around two thirds of patients with multiple injuries have severe thoracic injury abbreviated injury scale (AIS) ≥ 3 [1]. Mortality after trauma can be as high as 30% in patients with severe thoracic injury [2]. Although frequency and mortality are high, epidemiological studies are limited in the Nordic countries and without recent update, especially when evaluating risk factors for mortality following thoracic trauma.

When treating patients with multiple injuries, the evaluation of severity of thoracic trauma influences decision making in terms of first treatment and further clinical course [3]. Although there is plenty of literature on risk factors for mortality after trauma in general, little has been published on thoracic trauma specifically. Most thoracic trauma studies are analyses from trauma registries without supplementation from patient files, which makes the analysis of all relevant comorbidities and complications challenging [4–6].

One of the risk factors influencing mortality is injury severity, which is difficult to estimate due to a wide variety of scoring systems [7]. The scoring systems combining anatomical and physiological parameters seem to be the most suitable for severity assessment [7]. Next to injury severity, several other risk factors are thought to influence the clinical course of patients with thoracic trauma, such as age, sex, anticoagulant use, comorbidities and complications. An increase in the number of elderly patients with multiple trauma has been observed over the last years [8]. Hence it has been established that an age of 65 years and older is a predictive factor for mortality in patients with thoracic trauma [9, 10]. This is mainly due to diminished physiological reserves, inferior pre-injury functional capacities and age-related comorbidities [11].

Comorbidities influence mortality in thoracic trauma patients with odds ratios (OR) reported ranging from 1.2 in peripheral artery disease to as high as 6.6 in patients with metastatic cancer [5, 12, 13]. A systematic review about the influence of comorbidities on mortality is however lacking, and scoring systems of comorbidities have mostly been avoided.

Finally, complications may significantly alter the clinical course of thoracic trauma patients. Common complications as pneumonia and adult respiratory distress syndrome, are known causes of mortality following thoracic trauma [14]. There is limited literature on posttraumatic complications and mortality interaction in thoracic trauma. Additionally, to the best of our knowledge, no standardized scoring system for complications in thoracic trauma have been used yet. It has been shown that different injury mechanisms lead to different injury patterns [15]. Isolated thoracic injury, polytrauma with Injury Severity Score (ISS) ≥ 16 without Traumatic Brain Injury (TBI) and polytrauma with TBI as specific patient groups have differences in injury characteristics and could therefore theoretically demonstrate different predictive factors for mortality [16]. Therefore the aim of this study was to describe epidemiology and predictive factors for mortality following severe thoracic trauma in general and in relevant patient groups.

## Methods

### Study design

A retrospective study was performed on patients admitted to Haukeland University Hospital (HUS), a level one trauma center, between January 2009 and December 2018. Until December 2014, the patients were registered in the local hospital trauma registry and after that, in the Norwegian National Trauma Registry (NTR) [17]. The project was approved by the Regional Medical Ethics Committee in Norway under registration number 2017/293-8 [18]. Inclusion criteria were thoracic trauma with an AIS chest score of ≥ 3 and an age of 18 years or older at the day of the trauma. Patients not admitted directly to HUS and patients dead on arrival were excluded.

Baseline demographic information and clinical data were extracted from the HUS trauma registry and the NTR. The HUS trauma registry was prospectively maintained by dedicated trauma registrars which screen emergency patient admissions fulfilling predefined criteria in the local trauma registry until 2015. Thereafter, registration continued in the web-based NTR. In both registries, patients were labeled according to the AIS score (2005 version, update 2008) [19]. Patients were identified in the databases by selecting for thoracic injury AIS codes. Patient data was collected using a standardized case report form. Data on comorbidities, medication use and complications were supplemented by additional retrospective data collection. Patients with no record of comorbidities or medication use were distinguished from the cases where the absence of comorbidities was explicitly recorded. ISS, Glasgow Coma Scale (GCS), revised trauma score (RTS) and the trauma and injury severity Score (TRISS) were calculated at patient admission [16, 20, 21]. ISS ≥ 16 was applied as cutoff for polytrauma patients. Comorbidity data were scored according to the American Society of Anesthesiologists (ASA) score, Charlson comorbidity index (CCI) and comorbidity polypharmacy score (CPS) [22–24]. Complications were scored with the trauma adapted Clavien–Dindo scale (CDS) which classifies complications upon the most intensive therapy required complication treatment [25, 26]. To give an overview of all complications to a patient, the comprehensive complication index (CI) was calculated and analyzed [27].

### Statistical analyses

Primary end-point was 30-days mortality. The following factors and variables were analyzed for their contribution as a risk factor for in-hospital mortality. Age was categorized in four groups: 0–39, 40–59, 60–74 and 75 years and older. Comorbidities scored with CCI were categorized as 0, 1 and ≥ 2, according to common practice [28]. CPS was categorized as 0–9 and > 9 as appropriate [29] and also analyzed continuously. ISS was divided into three categories: minor 0–9, moderate 10–15, and severe with 16 and higher. RTS was stratified by odds of survival: low risk of death < 5% RTS ≥ 7.2, an intermediate risk of death ≥ 5% < 50% RTS ≥ 3.4 < 7.2, and a high risk of death ≥ 50% RTS < 3.4 [30]. TRISS was analyzed as a categorical variable in three categories with probability of survival (PS) 0–50, 51–75 and 76–100. ASA, Intensive care unit (ICU) and use of anticoagulants were regarded dichotomously. CDS was categorized in two groups, no or minor complications and severe CDS ≥ 3. Complications scored with CI were categorized as no or minor complications CI < 30, and severe CI ≥ 30 as previously reported [31]. The cut-off value for the CI was set at 30 to differentiate between major and minor complications based on the calculation of all possible comprehensive complication indices considering the worst complication as CDS grade two.

Categorical variables were presented using frequencies and percentages and tested using Pearson’s chi square test or Fischer exact test depending on the expected count > 5: Pearson, < 5: Fischer. Normally distributed variables were presented using means and standard deviations and tested using t tests and repeated measures ANOVAs. Non-Gaussian variables were presented as median and inter quartile range (IQR) and tested with the Kruskal–Wallis test.

Separate analyses for specific patient groups were conducted: (1) isolated thoracic injury with ISS < 16, (2) polytrauma without TBI which was defined as no AIS ≥ 3 head injury, and (3) polytrauma with AIS ≥ 3 head injury (TBI). Logistic regression analyses were executed with stepwise backward selection to identify risk factors for mortality following thoracic trauma. The *p*-value for inclusion of a variable in the logistic models was set at 0.157. As there were several variables that were used as a measure of the same factor, several different models were made to test each variable. To investigate a potential confounding influence of penetrating trauma, models with and without penetrating thoracic injuries were analyzed. The number of events (deaths, *n* = 4) in the isolated thoracic trauma patient group was too small to conduct a meaningful risk factor for mortality analysis with multivariate logistic regression. For each model, Hosmer–Lemeshow goodness of fit tests and areas under the Receiver Operating Characteristic (ROC) curve were calculated [32]. The best model was selected after calculating all the models, based on the Hosmer–Lemeshow test and the area under the ROC curve of the respective models. All statistical analyses were performed using StataCorp 2017 Stata Statistical Software: Release 15.

## Results

### Patient demographics

**Fig. 1:**
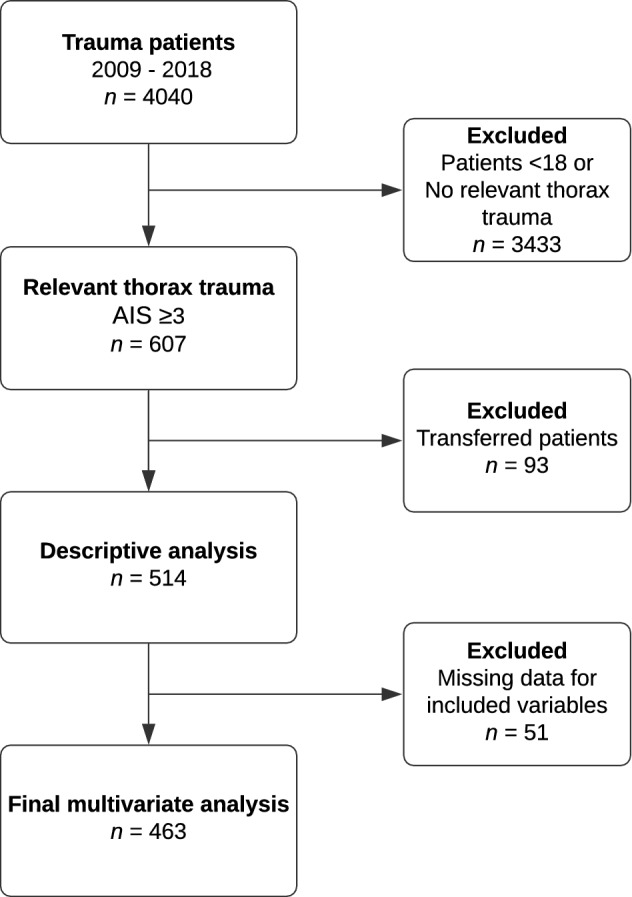
Flowchart of included and excluded patients for descriptive and regression analyses

**Fig. 2:**
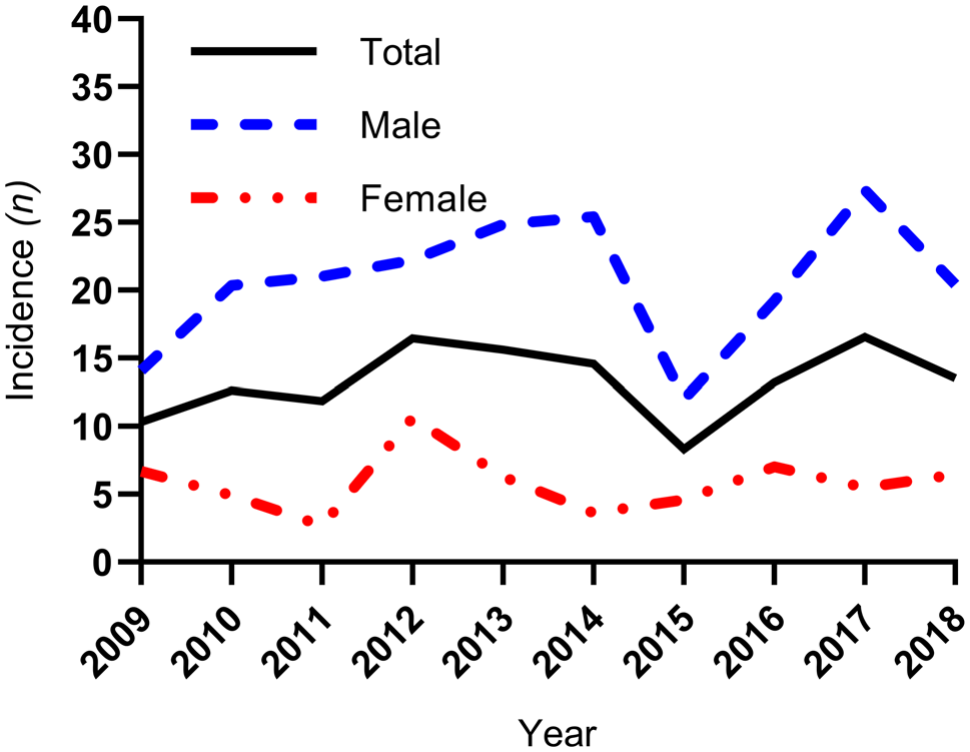
Annual incidence of thoracic trauma per 100,000 inhabitants in Haukeland University Hospital catchment area per year and sex

In total, 4042 patients were admitted during the inclusion period, of which 514 patients with thoracic injuries were included for analyses (Fig. 1). The mean annual incidence rate of severe thoracic trauma per 100,000 inhabitants remained the same during the study period, with a ten-year average of 13.3 cases (Fig. 2). Mean (SD) age was 51 [18] years and 78% were male (Table 1). Severe comorbidities according to CCI were present in 190 (40%) patients. Median (IQR) CPS was 0 (0–4). Most patients were in normal health status according to ASA classification (55%). Anticoagulants were used by 85 (17%) patients.

**Table 1:** Baseline characteristics of patients alive and deceased within 30 days after trauma

*n* (%)	Total (*n* = 514)	Patients alive at 30 days (*n* = 454)	Patients deceased within 30 days (*n* = 60)	*P* value*
Age (Mean, SD)	51 (19)	52 (18)	49 (21)	0.14
Male	402 (78)	357 (79)	45 (75)	0.52
Injury characteristics				
Blunt trauma^a^	471 (92)	420 (93**)**	51 (85)	0.07
ISS (Median, IQR)	17 (13–27)	17 (13–25)	34 (22–43)	** < 0.001**
Polytraumatized (ISS ≥ 16)	313 (61)	257 (57)	56 (93)	** < 0.001**
RTS^b^ (Median, IQR)	7.8 (7.1–7.8)	7.8 (7.6–7.8)	4.1 (2.6–7.8)	** < 0.001**
TRISS^c^ (Median, IQR)	1.0 (0.9–1.0)	1.0 (0.9–1.0)	0.5 (0.3–0.9)	** < 0.001**
Highest AIS – thorax				** < 0.001**
3	399 (78)	367 (81)	32 (53)	
4	86 (17)	70 (15)	16 (27)	
5	27 (5)	17 (4)	10 (17)	
6	2 (0)	0 (0)	2 (3)	
Other injuries				
AIS head ≥ 3	98 (19)	73 (16)	25 (42)	** < 0.001**
AIS face ≥ 3	10 (2)	8 (2)	2 (3)	0.41
AIS neck ≥ 3	7 (1)	2 (0)	5 (8)	** < 0.001**
AIS abdomen ≥ 3	52 (10)	39 (9)	13 (22)	**0.002**
AIS spine ≥ 3	34 (7)	31 (7)	3 (5)	0.59
AIS extremities ≥ 3	89 (17)	72 (16)	17 (28)	**0.016**
AIS external ≥ 3	2 (0)	1 (0)	1 (2)	0.09
AIS head ≥ 3				
Clinical characteristics				
ASA^d^ (Median, IQR)	1 (1–2)	1 (1–2)	2 (1–2)	0.15
CCI^e^ (Median, IQR)	1 (0–3)	1 (0–3)	1 (0–4)	0.26
CPS^f^ (Median, IQR)	0 (0–4)	0 (0–4)	1 (0–5)	0.55
Anticoagulants^g^	85 (18)	75 (17)	10 (17)	0.39
GCS^h^ ≤ 8	84 (16)	42 (9)	42 (70)	** < 0.001**
SBP on admission (Mean, SD)	127 (33)	131 (27)	95 (52)	** < 0.001**
LOS (Median, IQR)	7 (3–14)	9 (4–15)	1 (0–4)	** < 0.001**
ICU admission	213 (41)	184 (41)	29 (48)	0.25
Complications^i^				
≥ 1 complications during admission	189 (37)	147 (32)	42 (72)	** < 0.001**
No. of complications (Mean, SD)	0.6 (1)	0.5 (1)	0.9 (1)	** < 0.001**
Grade 1 *Resolves without intervention*	28 (6)	28 (6)	0 (0)	**0.05**
Grade 2 *Resolves with medical intervention*	87 (17)	83 (18)	4 (7)	**0.02**
Grade 3a *Operative treatment, local anesthesia*	24 (5)	23 (5)	1 (2)	0.24
Grade 3b *Operative treatment, general anesthesia*	24 (5)	24 (5)	0 (0)	0.07
Grade 4a *IC admission, single organ failure*	49 (10)	46 (10)	3 (5)	0.20
Grade 4b *IC admission, multi organ failure*	10 (2)	5 (1)	5 (8)	** < 0.001**
Grade 5a *Death, not actively treated*	13 (3)	0 (0)	13 (22)	** < 0.001**
Grade 5b *Death, actively treated*	24 (5)	0 (0)	24 (40)	** < 0.001**

Abbreviations: *SD* standard deviation, *IQR* Inter Quartile Range, *ISS* Injury Severity Score, *RTS* Revised Trauma Score, *TRISS* Trauma and Injury Severity Score, *AIS* Abbreviated Injury Scale, *ASA* American Society of Anesthesiologists classification, *CCI* Charlson Comorbidity Index, *CPS* Comorbidity Polypharmacy Score, *GCS* Glasgow Coma Scale, *SBP* Systolic Blood Pressure, *LOS* Length of Stay, *ICU* Intensive Care Unit

^*^Statistically significant results are bolded. Missing data: a: 2.0% b: 2.3% c: 4.3% d: 11.5% e: 4.7% f: 5.8% g: 6.8% h: 3.3% i: 2.3%. Percentages might not add up to 100% due to missing data

### Mechanism of injury

Most frequent mechanism of injury was road traffic accident (RTA) (Table 2). Overall, RTAs made up for 49% of mechanisms of injury with car crashes being the most frequent cause among RTAs (26%) followed by motorcycle crashes (9%), cycling accidents (7%) and injured pedestrians (3%). Falls made up for most of the remaining injuries (35%).

**Table 2:** Characteristics of subgroups on demographics, injury mechanism and mortality

*n*(%)	Total (*n* = 514)	Isolated thoracic trauma (*n* = 201)	Polytrauma without TBI (*n* = 215)	Polytrauma with TBI *(n* = *98*)
Age (Mean, SD)	51 (19)	53 (18)	52 (19)	47 (18)
Male	402 (78)	156 (78)	166 (77)	80 (82)
Injury characteristics				
ISS (Median, IQR)	17 (13–27)	11 (9–13)	22 (17–29)	29 (22–41)
TRISS (Median, IQR)^a^	1.0 (0.9–1.0)	1 (1–1)	0.9 (0.9–1)	0.8 (0.6–0.8)
Clinical characteristics				
CPS (Median, IQR)^b^	0 (0–4)	0 (0–4)	1 (0–4)	0 (0–3)
GCS ≤ 8 (Median, IQR)^c^				8 (3–15)
LOS (median, IQR)	7 (3–14)	5 (2–9)	10 (5–18)	11 (3–19)
Complications^d^				
No complication	325 (63)	167 (83)	120 (56)	38 (39)
Minor complication	66 (13)	15 (7)	35 (16)	16 (16)
Severe complication	123 (24)	19 (10)	60 (28)	44 (45)
No. of AIS ≥ 3 thoracic injuries				
1	338 (66)	167 (83)	113 (52)	58 (60)
2	116 (23)	27 (14)	62 (29)	27 (28)
3	47 (9)	7 (4)	32 (15)	8 (8)
> 3	13 (3)	0 (0)	8 (4)	5 (5)
Injury mechanism				
Car accident	134 (26)	54 (27)	55 (26)	25 (25)
Motorcycle	48 (9)	24 (12)	14 (7)	10 (10)
Cyclist	36 (7)	17 (8)	12 (6)	7 (7)
Pedestrian	15 (3)	4 (2)	6 (3)	5 (5)
Traffic other	19 (4)	6 (3)	10 (5)	3 (3)
Shot	2 (0.4)	2 (1)	0 (0)	0 (0)
Stabbed by sharp object	21 (4)	5 (2)	15 (7)	1 (1)
Stump object	23 (5)	9 (4)	9 (4)	5 (5)
Fall < 3 m	60 (12)	29 (15)	18 (8)	13 (13)
Fall > 3 m	122 (24)	39 (19)	55 (26)	28 (29)
Other	7 (1)	1 (1)	6 (3)	0 (0)
Unknown	27 (5)	11 (5)	15 (7)	1 (1)
Mortality				
30 day mortality	60 (100)	4 (100)	31 (100)	25 (100)
Cause of death				
Bleeding thorax	10 (17)	1 (25)	8 (26)	1 (4)
Bleeding abdomen	1 (2)	0 (0)	1 (3)	0 (0)
Multiple bleeding	6 (10)	1 (25)	2 (7)	3 (12)
Other bleeding	7 (12)	0 (0)	6 (19)	1 (4)
Asphyxia	3 (5)	0 (0)	3 (10)	0 (0)
Brain damage	18 (30)	0 (0)	0 (0)	18 (72)
Organ failure	8 (13)	1 (25)	7 (23)	0 (0)
Other	2 (3)	0 (0)	1 (3)	1 (4)
Unknown	5 (8)	1 (25)	3 (10)	1 (4)

Missing data: a: 4.3% b: 5.8 c: 3.3% d: 2.3%. Percentages might not add up to 100% due to missing data

Abbreviations: *SD* standard deviation, *IQR* Inter Quartile Range, *ISS* Injury Severity Score, *TRISS* Trauma and Injury Severity Score, *CPS* Comorbidity Polypharmacy Score, *GCS* Glasgow Coma Scale, *LOS* length of stay

### Injury characteristics, treatment and complications

**Fig. 3:**
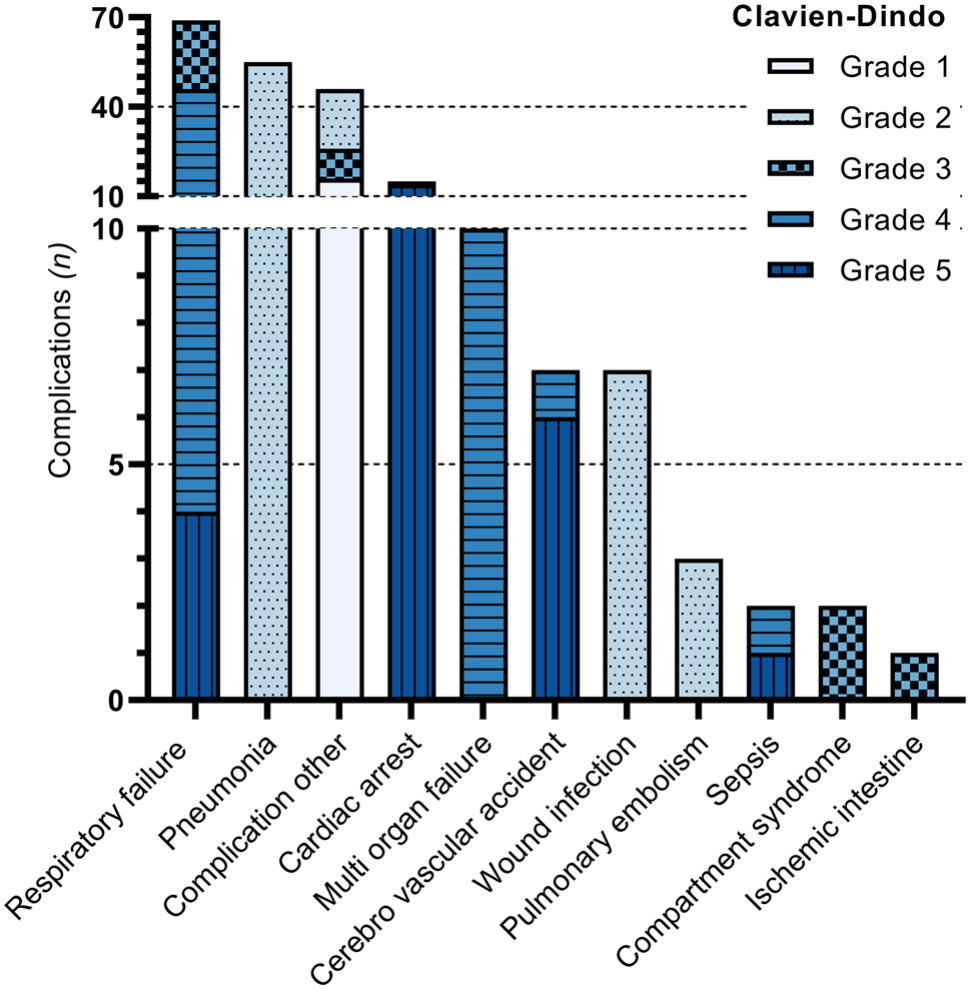
Frequencies and nature of severe complications. *CDS grade 1* requires no treatment, *CDS grade 2* requires pharmalogical treatment, *CDS grade 3* requires surgical treatment, *CDS grade 4* requires intensive care, *CDS grade 5* death ()

**Fig. 4:**
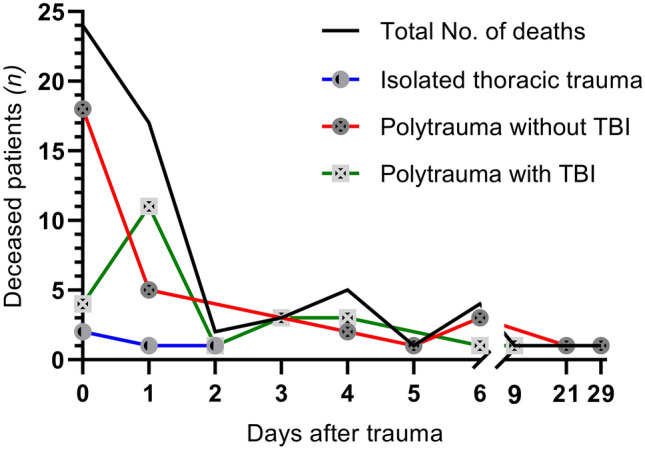
Temporal distribution of mortality. Abbreviations: *TBI* traumatic brain injury

Median ISS was 17 (13–27) and median RTS was 7.8 (7.1–7.8) (Table 1). Most commonly co-occurring injuries were head and abdominal injuries. Rib fractures were the most prevalent thoracic injury, occurring in 426 (83%) patients (Table 3). In 19 (4%) patients an emergency thoracotomy was performed. In 126 (25%) patients a chest tube was placed. Complications occurred in 189 (37%) patients (Table 4, Fig. 3). Most common severe complication (CDS ≥ 3) was respiratory failure in 70 (14%) patients. Within the first 24 h after the accident 24 (5%) patients died (Fig. 4). Sixty patients (12%) died within 30 days after the accident. Three hundred thirteen (61%) patients were polytrauma casualties.

**Table 3:** Injury and treatment details

*n* (%)	Total (*n* = 514)	Patients alive at 30 days (*n* = 454)	Patients deceased within 30 days (*n* = 60)	*P* value*
Most common thoracic injuries				
Lung contusion	226 (44)	194 (43)	32 (53)	0.12
Pneumothorax	196 (38)	173 (38)	23 (38)	0.97
Flail chest	72 (14)	56 (12)	16 (27)	**0.003**
Rib fracture	426 (83)	383 (84)	43 (72)	**0.01**
Hematothorax	40 (8)	27 (6)	13 (22)	** < 0.001**
Lung laceration	13 (3)	5 (1)	8 (13)	** < 0.001**
Sternal fracture	80 (16)	68 (15)	12 (20)	0.31
Thoracic vessel injury	17 (3)	7 (2)	10 (17)	** < 0.001**
Cardiac injury	29 (6)	19 (4)	10 (17)	** < 0.001**
First emergency procedure^a^				
None	425 (85)	398 (88)	30 (50)	** < 0.001**
Damage control thoracotomy	19 (4)	6 (1)	13 (22)	** < 0.001**
Damage control laparotomy	26 (5)	18 (4)	8 (13)	**0.002**
Extraperitoneal pelvic packing	3 (1)	1 (0)	2 (3)	**0.004**
Revascularisation of an extremity	1 (0)	1 (0)	0 (0)	
Intervention radiology	3 (1)	3 (1)	0 (0)	
Craniotomy	3 (1)	3 (1)	0 (0)	
Intracranial pressure gauge placement	19 (4)	14 (3)	5 (8)	0.06
Chest tube placement^b^				
Emergency department	99 (19)	84 (19)	15 (25)	0.23
No drain	367 (71)	336 (74)	31 (52)	** < 0.001**
Pre-hospital drain	27 (5)	15 (3)	12 (20)	** < 0.001**

^*^Statistically significant results are bolded. Missing data: a: 1% b: 4.1%. Percentages might not add up to 100% due to missing data

**Table 4:** Number of patients with complications and their nature

*n (%)*	Total (*n* = 514)	Patients alive at 30 days (*n* = 454)	Patients deceased within 30 days *(n* = 60)	*P* value*
CD grade 1				
Complication other^†^	16 (3)	16 (4)	0 (0)	0.14
CD grade 2				
Pneumonia	55 (11)	52 (11)	3 (5)	0.13
Pulmonary embolism	3 (1)	3 (1)	0 (0)	0.53
Wound infection	7 (1)	7 (2)	0 (0)	0.33
Complication other	20 (4)	19 (4)	1 (2)	0.34
CD grade 3a				
Respiratory failure (chest tube next day)	14 (3)	14 (3)	0 (0)	0.17
Complication other^†^	8 (2)	7 (2)	1 (2)	0.94
CD grade 3b				
Respiratory failure (chest tube next day)	9 (2)	9 (2)	0 (0)	0.27
Compartment syndrome	2 (0)	2 (0)	0 (0)	0.60
Ischemic intestine	1 (0)	1 (0)	0 (0)	0.72
Complication other^†^	2 (0)	2 (0)	0 (0)	0.60
CD grade 4a				
Respiratory failure	42 (8)	39 (9)	3 (5)	0.34
Sepsis	1 (0)	0 (0)	1 (2)	0.12
CVA	1 (0)	1 (0)	0 (0)	0.88
Cardiac arrest	1 (0)	0 (0)	1 (2)	0.12
CD grade 4b				
Multi organ failure	10 (2)	6 (1)	4 (7)	**0.005**
CD grade 5a				
Respiratory failure	3 (1)	0 (0)	3 (5)	**0.002**
CVA	4 (1)	0 (0)	4 (7)	** < 0.001**
Cardiac arrest	2 (0)	0 (0)	2 (3)	**0.01**
CD grade 5b				
Respiratory failure	1 (0)	0 (0)	1 (2)	**0.**12
Sepsis	1 (0)	0 (0)	1 (2)	**0.**12
CVA	2 (0)	0 (0)	2 (3)	**0.01**
Cardiac arrest	12 (2)	0 (0)	12 (20)	** < 0.001**

Abbreviations: *CD* Clavien–Dindo, *CVA* Cerebro Vascular Accident

^*^Statistically significant results are bolded. ^**†**^Other complications that occurred with *n* < 5. Missing data: 2.3%. Percentages might not add up to 100% due to missing data

### Risk factors for mortality following thoracic trauma

The analyses showed that female sex, more than nine comorbidities and medication use combined, a TRISS with a PS of lower than 50% and all the complications of a patient with a CI ≥ 30 were associated with an adverse outcome after trauma (Table 5). The final model (Hosmer–Lemeshow goodness of fit test (*p* = 0.4), area under ROC curve (0.93) consisted of the predictors mentioned above. Females had a 2.7 times higher risk of dying compared to male victims (OR 2.7, *p* = 0.04). Having more than nine comorbidities and medication use combined lead to nearly five times higher risk of dying (OR 4.8, *p* = 0.01). TRISS was also a strong risk factor for mortality where patients with a PS of 0 to 50%, having a 44 times higher risk of dying (OR 44, *p* < 0.001). Finally, patients with severe complications measured by CI, resembling a minimal complication of CDS grade ≥ 3, were 12.5 times more likely to die compared to those having a mild or no complication (OR 12.5, *p* < 0.001). Anticoagulants were no risk factors for mortality in this study. No confounding effect of penetrating thoracic injuries was observed. The analyses with penetrating thoracic trauma excluded showed the same significant results (online resource supplementary Table 1). Characteristics of the patients included and excluded in the risk factor for mortality analyses are given in Table 6.

**Table 5:** Multivariable analysis for mortality

Factor and variable	Odds ratio	95% CI for odds ratio Upper–Lower	*P *value*
Female sex	**2.7**	**1.0–7.1**	**0.04**
TRISS 75–100	Ref	–	–
TRISS 50–75	2.5	0.6–10.6	0.22
TRISS 0–50	**43.9**	**14.1–136.9**	** < 0.001**
CPS > 9	**4.8**	**1.4–15.9**	**0.01**
CI ≥ 30	**12.5**	**4.8–32.5**	** < 0.001**

Abbreviations: *TRISS* Trauma and Injury Severity score, *C.I* Comprehensive Complication Index, *CPS* Comorbidity Polypharmacy Score, *CI* Confidence Interval

^*^Statistically significant results are bolded

**Table 6:** Demographics of patients included and excluded in the final model and excluded transferred patients

*n* (%)	Patients in model *(n* = 463)	Patients excluded from final regression model due to missing data (*n* = 51)	Transferred patients that were excluded from the study *(n* = *93)*	Total AIS ≥ 3 thorax trauma admitted to HUS (*n* = 607)
Deaths	39 (8)	21 (41)	7 (8)	67 (11)
Median ISS (IQR)	17 (13–25)	29 (19–38)	25 (18–36)	19 (13–29)
Mean Age (SD)	52.5 (19)	43.0 (18)	52.3 (19)	51.6 (19)
Sex (male)	360 (78)	42 (82)	77 (80)	477 (79)

Abbreviations: *ISS* Injury Severity Score

### Analyses of specific patient groups

Subgroup analyses were conducted for three specific groups: 201 isolated thoracic trauma patients, 215 polytrauma without TBI and 98 polytrauma with TBI patients (Table 2). Mean age (SD) of polytrauma patients with TBI was significantly lower (47, 19) than polytrauma without TBI patients (52, 19) and isolated thoracic trauma patients (53, 18), (*p* < 0.001). There were no differences in sex distribution or comorbidities between subgroups. Low-energy falls < 3 m were relatively frequent (15%) in isolated thoracic trauma compared to the other patient groups. High energy falls > 3 m were more often the causative mechanism in both polytrauma groups. Median (IQR) ISS was significantly higher in polytrauma without TBI (22, 17–29) and polytrauma with TBI (29, 22–41) than in isolated thoracic injury (11, 9–13), (*p* < 0.001). In the polytrauma without TBI group the highest number of AIS ≥ 3 thoracic injuries were observed with 102 (48%) patients with two or more AIS ≥ 3 thoracic injuries. There were 44 (45%) patients with severe complications in the polytrauma with TBI group. This was significantly higher than the 60 (28%) patients in the polytrauma without TBI group and the 19 (10%) patients in the isolated thoracic trauma group (*p* < 0.001). The highest mortality was observed in the polytrauma with TBI group with 25 (25%) deaths. In polytrauma without TBI the highest number of fatalities occurred within 24 h after trauma with 18 (58%) deaths, while the majority of deaths in polytrauma with TBI occurred on the second day with 11 (45%) deaths (Fig. 4). Main cause of death in the polytrauma without TBI group was exsanguination. In the polytrauma with TBI group main cause of death was brain injury. Risk factor for mortality analyses for specific subgroups did not reveal other risk factors for mortality than in the total thoracic trauma cohort in this study. Female sex was no risk factor for mortality in polytrauma without TBI (online resource supplementary Tables 2, 3).

### Data quality

Data was missing in the following variables: injury mechanism (5.3%), nature of the trauma mechanism (2.0%), GCS (3.3%), RTS (2.3%), TRISS (4.3%) ASA (11.5%) CCI (4.7%) CPS (5.8%) anticoagulants (6.4%), first emergency procedure (1.0%), chest tube (4.1%) complications (2.3%). Missing data were likely not to be random; mortality was significantly higher in the group of patients with missing data on any of the variables.

## Discussion

The present study aimed to clarify epidemiological data, injury characteristics, in-hospital outcomes and risk factors for mortality following severe thoracic trauma at a level one trauma center. Based upon a cohort of 514 adult patients, this study demonstrated that more than nine comorbidities and medication use combined, TRISS, female sex, as well as severe complications, were significant risk factors for mortality following severe thoracic trauma. Analyses of specific patient groups did not demonstrate other risk factors.

### Epidemiology of thoracic trauma in Western Norway and mortality

The average of 51 treated patients with severe thoracic injury at HUS per year is roughly comparable to other hospitals that have studied thoracic trauma patients [6, 9]. The incidence rate of polytraumatized patients with severe thoracic trauma in Western Norway (8/100,000 inhabitants) was comparable to that of the Netherlands (9/100,000 inhabitants). The percentage of car accidents as cause of severe thoracic trauma with polytrauma in HUS (50%), was relatively low compared to the university hospital of Helsinki (58%) and German hospitals (64%) [6]. It is known that Norway is one of the safest countries with regards to traffic and has one of the lowest traffic accident mortality rates in the world [33]. Mortality after severe thoracic trauma in polytrauma varies within the current literature, ranging from 10 to 18%, with no cohorts exactly matching this study [4–6, 9, 34]. In this study, overall mortality in the polytrauma group, a cohort similar with the aforementioned literature, was relatively high with 18%. There were no general trends in different age, ISS or comorbidities if reported in those studies compared to HUS. Future studies are required to determine the cause of this difference in mortality.

Mortality in this study was not according to the classical trimodal pattern of trauma deaths by Trunkey et al*.* [35]. The classical model describes deaths on scene, these were not included in this study, early deaths within 6 h after trauma, and a third peak of late deaths three to 4 weeks after trauma. This late peak was not observed in this study. As shown before, it is hypothesized that the late peak of deaths due to sepsis and respiratory insufficiency does not occur due to improvements in health care systems [36]. In this study mortality in polytrauma without TBI was the highest in the first 24-h after trauma due to exsanguination. In polytrauma with TBI the highest mortality was later than 24-h after trauma, and the main reason for death in this subgroup was brain injury. Deaths due to exsanguination in this study show the second peak of early deaths of the classical trimodal pattern of trauma deaths, and primary brain injury deaths do not, with most of brain injury deaths occurring later than 24-h after trauma. This is in line with current literature [36–38].

### Comorbidity as risk factor for mortality

Comorbidities that have been shown to predict mortality after trauma are: hepatic and renal disease, congestive heart failure, hypertension, pulmonary disease, diabetes, malignancy, obesity, peripheral artery disease and neurologic disorders [5, 39–43]. This study demonstrates patients having more than nine comorbidities and medication use combined measured by CPS, to have a considerably higher mortality risk compared to patients with relatively few or no comorbidities (OR 4.8, *p* = 0.011). This was demonstrated using a relatively novel, standardized instrument for quantifying comorbidities. A (geriatric) trauma study by Nossaman et al. in 2018 reported CPS to be a risk factor for mortality, although patients with a higher CPS demonstrated lower mortality risk [44]. The authors hypothesized that patients with known comorbidities and the respective medication use may have returned to a better physiologic conditions than their peers with unknown comorbidities and medication (a low measured CPS). These patients might have suffered from the same comorbidities but were not treated and therefore had a higher mortality risk. We hypothesize that the number of care avoiders in Norway is low due to a free health care system while as much as 45% of US citizens delays or avoids medical care due to costs [45]. Hence, Norwegian patients with a high CPS may be less healthy than Norwegian patients with a low CPS, they may thus have a higher mortality risk.

It has been debated whether higher mortality in older patients is caused by ageing itself or rather by more comorbidities in elderly patients [41, 46]. The present study demonstrates that comorbid conditions independently predict mortality. This is supported by Milzman et al. who showed that the effect of comorbidities on mortality in trauma patients proved consistent over all age and injury severity groups [40]. The main reason for adverse outcomes in patients with severe comorbidities is the reduced physiological reserve that is caused by comorbidities [11].

### CPS as a predictor of mortality

In the present study, CPS as a measure of comorbidities, appeared to be the strongest predictor of mortality, even stronger than the CCI. The literature on this topic is contradicting, since both CPS and CCI have previously shown to predict mortality in trauma [47, 48]. However, to the best of our knowledge, CPS and CCI have not been compared. In our study, the number of comorbidities combined with medication use is a more accurate predictor of mortality than the comorbidities included in the CCI. This suggests that there are other relevant comorbidities influencing survival that are not included in the CCI. With this result, we would like to emphasize, that the number of comorbid conditions and medication use is a helpful tool to estimate mortality risk in thoracic trauma patients.

### Injury severity

Several trauma outcomes and injury severity scores are related to injury severity. In the present study injury severity in thoracic trauma is best measured by TRISS. The TRISS value is based upon registered ISS and RTS as well as age and type of trauma, hence giving an estimation of probability of mortality following trauma. It is well known that the anatomical severity of the injury combined with the physiological burden to the body is the best measure of injury severity and therefore the strongest predictor for mortality after trauma [49, 50].

### Complications

This is, to our knowledge, the first study that provides an overview of all complications that occur after severe thoracic trauma with a standardized method. We find the overall complication rate of thoracic trauma to be comparable with complication rates mentioned in recent studies on rib fractures in polytrauma patients, both in polytrauma patients in general as well as in geriatric [5, 8, 51]. This study emphasizes the high risk of pulmonary complications occurring after thoracic trauma, and the higher mortality in this patient group. Pneumonia and respiratory failure were frequently observed in our material. Also, patients with a CI ≥ 30, representing severe complications of CDS grade 3 and higher were more likely to die (OR 12.5, *p* < 0.001). Of CDS grade 3 and higher complications, 65% were pulmonary complications. It has been observed that respiratory complications are a frequent cause of death in older patients [8, 14]. The multivariate models with CI were marginally better than the models with CDS, both were therefore risk factors for mortality in this study. The number of severe complications was significantly higher in the polytrauma with TBI group than in isolated thoracic trauma and polytrauma without TBI subgroups. This result can be explained by polytrauma with TBI being the most severely injured patient group with the highest ISS, and deaths due to brain injury occur relatively late compared to exsanguination, therefore there is more time to develop complications.

### Age and use of anticoagulants

An unexpected finding in the present study was that age and anticoagulants were no independent risk factors for mortality. In none of the analyzed models, age was an independent risk factor for mortality. Advancing age leads to a higher mortality and has been found to be a risk factor for mortality in a large number of previous studies [4, 9, 12, 43, 52, 53]. The exact relationship between mortality, age and comorbidities remains debated. The present and other studies suggest that comorbidities accompanying advanced age contribute to mortality, as opposed to age alone [54].

Anticoagulant use predicts mortality after head trauma [55]. However, anticoagulant use did not emerge as predictor of mortality among our patients. The sample size may have been too small to demonstrate an effect of higher bleeding tendency on mortality. In addition, anticoagulant use also means that the patient has comorbidities. The models with CPS included, appear to be a stronger predictor for mortality than anticoagulant use alone. These effects could have caused anticoagulants not to become a significant predictor in our material.

Potential limitations of the present study arise mostly from its retrospective character. Underestimation of effects could be caused due to missing data on comorbidities, medication use and complications in patients that die within the first 24 h after the accident. Patients who died on scene are excluded. In Hordaland county, of which HUS is both the local and level one trauma center, 72% of patients who die after trauma, die at the scene or before arrival at a hospital [56]. This may cause an underestimation of the contribution of the investigated factors on mortality. Subgroups were relatively small and this may be the reason that no other risk factors were found in the subgroup analyses. Complications were defined as all adverse events graded by CDS occurring on the ward or in the ICU in patients after the initial stabilization and treatment. This could be a limitation in the risk factor for mortality analyses in patients who died in the first 48 h after trauma. There is a partial similarity between injury severity and complications. The significant risk factor complications might have become significant partially by the mortality in the first 48 h after trauma due to the severity of the injury.

In this study severe complications CD ≥ 3 were a significant risk factor for mortality and these complications occurred in 23% of all patients. It is important to be aware of patients at risk of developing a severe complication. These patients should be monitored intensively and treated aggressively as soon as complications occur to prevent further health deterioration. To the best of our knowledge, only one study on risk factors for complications in thoracic trauma exists, focusing solely on pneumonia [13]. Future studies should focus on identifying risk factors for complications following thoracic trauma, with subgroup analyses of patients who survive the first 48 h, to gain insight in patients at risk and in need of active trauma level one treatment.

## Conclusions

The present study demonstrates thoracic injury to be common within the Haukeland University Hospital catchment area, causing relatively high mortality. In a model with multiple known risk factors, more than nine comorbidities and medications combined, TRISS, female sex, and severe complications predict mortality after thoracic trauma. In analyses of patient groups with polytrauma and without TBI, and polytrauma with TBI, no significant other risk factors were found. This study highlights the importance of gathering information about the patient and the injury mechanism as early as possible in the trauma evaluation process and early care when severe thoracic injury is suspected. Current findings may help to recognize patients at risk of an adverse outcome and prevent complications.

## Supplementary Information

Below is the link to the electronic supplementary material.

Supplementary file1 (DOCX 27 KB)

## Data Availability

The data that support the findings of this study are available from the University of Bergen, Haukeland University Hospital, Western Norway Trauma Center, but restrictions apply to the availability of these data, which were used under license for the current study, and so are not publicly available. Data are however available from the authors upon reasonable request and with permission of the Haukeland University Hospital.

## Abbreviations

AIS: Abbreviated injury scale
OR: Odds ratio
ISS: Injury severity score
TBI: Traumatic brain injury
HUS: Haukeland University Hospital
NTR: National Trauma Registry
GCS: Glasgow coma scale
RTS: Revised trauma score
TRISS: Trauma and injury severity score
ASA: American Society of Anesthesiologists
CCI: Charlson comorbidity index
CPS: Comorbidity polypharmacy score
CDS: Clavien–Dindo scale
CI: Comprehensive complication index
PS: Probability of survival
ICU: Intensive care unit
IQR: Inter quartile range
ROC: Receiver operating characteristics
RTA: Road traffic accident
