# Supplementary material for: Comorbidities, injury severity and complications predict mortality in thoracic trauma
Source: Eur J Trauma Emerg Surg. 2022 Dec 17;49(2):1131–43. doi: 10.1007/s00068-022-02177-6 (PMC10175434; doi:10.1007/s00068-022-02177-6)
Supplement: Supplementary file 1 — Supplementary file1 (DOCX 27 KB) [file 68_2022_2177_MOESM1_ESM.docx]

Article title **Comorbidities, injury severity and complications predict mortality in thoracic trauma**

**Journal name: European Journal of Trauma and Emergency Surgery**

Anne T. Fokkema^a^; Bergros K. Johannesdottir^b, d^; Klaus Wendt^a^; Rune Haaverstad^b, c, e^ ; Inge H. F. Reininga^a^; Thomas Geisner^f^

^a^University of Groningen, University Medical Center Groningen, Department of Trauma Surgery, Groningen, the Netherlands

^b^Haukeland University Hospital, Department of Vascular Surgery, Bergen, Norway

^c^University of Bergen, Haukeland University Hospital, Section of Cardiothoracic Surgery, Department of Heart Disease, Bergen, Norway

^d^University of Iceland, Reykjavik, Iceland

^e^University of Bergen, Institute of Clinical Science, The Medical Faculty, Bergen, Norway

^f^Haukeland University Hospital, Western Norway Trauma Center, Bergen, Norway

**Corresponding author:**

Anne T. Fokkema, MD

University of Groningen, University Medical Center Groningen

Department of Trauma Surgery

P.O. Box 30.001, Hanzeplein 1, 9700RB Groningen, The Netherlands

Email: [a.t.fokkema@umcg.nl](mailto:a.t.fokkema@umcg.nl)

Phone: +31503612876

ORCID ID: 0000-0002-2797-3465

Supplementary tables 1a, 1b: results of multivariable analysis for risk factors for mortality without penetrating thoracic trauma.

Supplementary tables 2a, 2b, 3a, 3b

Results of risk factor for mortality analysis in specific subgroups.

| **Supplementary Table 1a: Multivariable analysis for mortality without penetrating thoracic trauma** | | | | | | | |
| --- | --- | --- | --- | --- | --- | --- | --- |
| **Factor and variable** | |  | **Odds ratio** |  | **95% CI for odds ratio**  **Upper - Lower** |  | **P-value*** |
|  | **Female gender** |  | **3.1** |  | **1.2-8.4** |  |  |
|  | TRISS 75-100 |  | Ref |  | - |  | **0.02** |
|  | TRISS 50-75 |  | 2.2 |  | 0.5-9.5 |  | - |
|  | **TRISS 0-50** |  | **28.5** |  | **8.6-94.6** |  | 0.31 |
|  | **CPS > 9** |  | **5.6** |  | **1.6-19.4** |  | **<0.001** |
|  | **CI ≥ 30** |  | **15.2** |  | **5.5-42.5** |  | **0.01** |
| *Statistically significant results are bolded. Abbreviations: TRISS, Trauma and Injury Severity score; C.I, Comprehensive Complication Index; CPS, Comorbidity Polypharmacy Score;, CI, Confidence Interval. | | | | | | | |

| **Supplementary Table 1b: Demographics of patients included and excluded in the final model without penetrating thoracic trauma.** | | | | |  |
| --- | --- | --- | --- | --- | --- |
| ***n (%)*** |  | **Patients in model**  ***(n* = 423)** |  | **Patients excluded from final regression model***  **(*n* = 91)** |  |
|  |  |  |  |  |  |
| Deaths |  | 35 (8) |  | 25 (28) |  |
| Median ISS (IQR) |  | 17 (13-25) |  | 25 (16-36) |  |
| Mean Age (SD) |  | 54 (18) |  | 42 (17) |  |
| Gender (male) |  | 323 (76) |  | 79 (87) |  |

*due to missing data or penetrating trauma

| **Supplementary Table 2a: Multivariable analysis for mortality in subgroup polytrauma without TBI.** | | | | | | |
| --- | --- | --- | --- | --- | --- | --- |
| **Factor and variable** |  | **Odds ratio** |  | **95% CI for odds ratio**  **Upper - Lower** |  | **P-value*** |
| **Final multivariate model** |  |  |  |  |  |  |
| TRISS 75-100 |  | Ref |  |  |  |  |
| TRISS 50-75 |  | 3.3 |  | 0.8–13.9 |  | 0.1 |
| TRISS 0-50 | | Removed in the model due to direct correlation with mortality | | | | |
| CPS > 9 |  | 5.3 |  | 0.8– 33.3 |  | 0.07 |
| **CD ≥ 3** |  | **17.5** |  | **3.5-88.3** |  | **0.001** |
| *Statistically significant results are bolded. Abbreviations: TRISS, Trauma and Injury Severity score; CD, Clavien Dindo; CPS, Comorbidity Polypharmacy Score;, CI, Confidence Interval. | | | | | | |

| **Supplementary Table 2b: patients included and excluded in subgroup analysis polytrauma without TBI** | | | |
| --- | --- | --- | --- |
| ***n (%)*** | **Patients in model**  **(*n* = 184 *)*** |  | **Excluded patients***  **(*n* = 31)** |
| Deaths | 14 (8) |  | 17 (55) |
| Median ISS (IQR) | 22 (17-26) |  | 29 (19-38) |
| Mean Age (SD) | 54 (19) |  | 42 (15) |
| Sex (male) | 141 (77) |  | 25 (81) |
| Abbreviations: ISS, Injury Severity Score. *Excluded patients due to missing data or estimability. | | | |

| **Supplementary Table 3a: Multivariable analysis for mortality in subgroup polytrauma with TBI.** | | | | | | |
| --- | --- | --- | --- | --- | --- | --- |
| **Factor and variable** |  | **Odds ratio** |  | **95% CI for odds ratio**  **Upper - Lower** |  | **P-value*** |
| Female sex |  | 3.7 |  | 0.9-7.1 |  | 0.08 |
| TRISS 75-100 |  | Ref |  | - |  | - |
| TRISS 50-75 |  | 1.9 |  | 0.6-10.6 |  | 0.61 |
| TRISS 0-50 |  | 17.9 |  | 14.1-136.9 |  | 0.06 |
| CPS > 9 |  | 6.0 |  | 1.4-15.9 |  | 0.14 |
| **CI ≥ 30** |  | **13.9** |  | **4.8-32.5** |  | **0.001** |
| *Statistically significant results are bolded. Abbreviations: TRISS, Trauma and Injury Severity score; C.I, Comprehensive Complication Index; CPS, Comorbidity Polypharmacy Score;, CI, Confidence Interval. | | | | | | |

| **Supplementary Table 3b: patients included in analysis subgroup polytrauma with TBI.** | | | |
| --- | --- | --- | --- |
| ***n (%)*** | **Patients in model (*n* = 80*)*** |  | **Excluded patients****  **(*n* = 18)** |
| Deaths | 17 (21) |  | 8 (45) |
| Median ISS (IQR) | 29 (22-40) |  | 36 (34-45) |
| Mean Age (SD) | 48 (18) |  | 41 (18) |
| Sex (male) | 63 (79) |  | 17 (94) |
| Abbreviations: ISS, Injury Severity Score. *Excluded patients due to missing data or estimability. | | | |
